# Investigating the association between infertility and psychological distress using Australian Longitudinal Study on Women's Health (ALSWH)

**DOI:** 10.1038/s41598-022-15064-2

**Published:** 2022-06-25

**Authors:** Tanmay Bagade, Kailash Thapaliya, Erica Breuer, Rashmi Kamath, Zhuoyang Li, Elizabeth Sullivan, Tazeen Majeed

**Affiliations:** 1 College of Health, Medicine and Wellbeing, The University of Newcastle (UON), University Drive, Callaghan, NSW 2308 Australia; 2 Samatva Wellness Center, Noida, India; 3 Registry of Senior Australians (ROSA), South Australian Health and Medical Research Institute (SAHMRI), North Terrace, Adelaide, SA 5000 Australia

**Keywords:** Epidemiology, Psychology, Health care, Risk factors

## Abstract

Infertility affects millions of people globally. Although an estimated 1 in 6 couples in Australia are unable to conceive without medical intervention, little is known about the mental health impacts of infertility. This study investigated how infertility impacts the mental health of women. The study used nationally representative Australian Longitudinal Study on Women's Health (ALSWH) data. We analysed data from survey periods 2–8 conducted every three years between 2000 and 2018 for 6582 women born in 1973–78. We used a Generalised Equation Modelling (GEE) method to investigate the association of primary, secondary and resolved fertility status and psychological distress over time. Multiple measures were used to measure psychological distress: the (1) the mental health index subscale of the 36-item short form survey (SF-36), (2) the Center for Epidemiological Studies Depression Scale (CESD-10), (3) the Goldberg Anxiety and Depression Scale (GADanx) anxiety subscale; and a (4) composite psychological distress variable. About a third (30%) of women reported infertility at any of the survey rounds; a steady increase over 18 years from 1.7% at round 2 to 19.3% at round 8. Half of the women reporting primary or secondary infertility reported psychological distress, with the odds of having psychological distress was higher in women reporting primary (odds ratio (OR) 1.24, 95% confidence interval (CI) 1.06–1.45), secondary (OR 1.27, 95% CI 1.10–1.46) or resolved infertility (OR 1.15, 95% CI 1.05–1.26) compared to women reporting normal fertility status. Women with partners, underweight or higher BMI, smoking, and high-risk alcohol use had higher odds of psychological distress, whereas women in paid work had significantly lower odds of psychological distress (*p* < 0.001). Diabetes, high blood pressure, asthma, and other chronic physical illness were independently associated with higher odds of psychological distress. Infertility has a significant impact on mental health even after it is resolved. Frequent mental health assessment and a holistic approach to address the lifestyle factors should be undertaken during the treatment of infertility.

## Introduction

In 2018, an estimated 49–180 million couples globally were suffering from infertility^1^, defined by the World Health Organisation as a disease that affects the couple's inability to achieve conception despite regular and unprotected intercourse for over 12 months^2^. The estimated Global Burden of Disease due to female and male infertility has increased since 1990^3^. The global age-standardised Disability Adjusted Life Years due to female infertility increased by 15.83% from 1990 to 2017, compared to an 8.84% increase in male infertility^3^. In developed countries, infertility prevalence is 15%^4^, whereas, in Australia, the prevalence is estimated to be 17% for women aged between 28 and 33 years^5^. While the prevalence is unknown in the First Nations people of Australia, Gilbert et al. noted a higher incidence of risk factors associated with infertility^6^. Despite having a higher total fertility rate, the First Nations people in Australia may be disproportionately affected by infertility^6^.

Infertility is defined as primary when a pregnancy is never achieved or secondary when a minimum of one pregnancy is attained, but the individuals have difficulties achieving another conception^2^. It may take several years for couples with infertility to achieve a healthy full-term pregnancy, and some do not achieve this even with intervention^7^. Individuals often only seek infertility-related services when they strongly feel that embracing parenthood is a required social role for them at that stage of their life; therefore, the individual's definition of infertility might be different from what is understood in the literature^8^.

The process of conceiving and having a healthy child can be a challenging and stressful journey, which can further make the individuals feel powerless^9^. A systematic review on quality of life in infertility patients revealed that individuals could spend an average of 8.22 years with infertility^10^. Individuals scored significantly lower on mental health, social functioning and emotional behaviour and failed treatment was associated with lower quality of life^10^. Jacob et al. reported that women seeking infertility treatment show a 16% higher level of psychological distress than those without infertility^11^. Likewise, several other studies conducted in the USA, Finland, Norway have shown an association between infertility and adverse mental health outcomes^12–14^. Berg and Wilson summarised the cluster of anxiety, irritability, depression, blaming the self, lethargy, loneliness, and vulnerability as common infertility-related mental health symptoms^15^. The psychological effects of infertility are not limited to short-term impacts but can affect the long-term mental health and wellbeing of couples. Although infertility can also result in poor mental health outcomes among men^16^, women often experience more psychological distress over time^17^. Women who elect not to have children or are childless due to fertility issues reported poorer social wellbeing and emotional health than the overall female population of Australia^18–20^.

The literature on infertility is disproportionately skewed towards clinical research related to causes of infertility, diagnosis, or Artificial Reproductive Techniques (ART), and lesser on the anthropological, population and public health aspects^21^. Previous studies (nationally and internationally) have looked at cross-sectional data to assess the association between infertility and mental health^17,22–24^. However, the study findings of previous studies are either qualitative or limited to a specific facility or have smaller homogenous sample sizes. Furthermore, due to the lengthy duration of infertility diagnosis and treatment, the association of fertility status with mental health outcomes cannot be studied through short-term cross-sectional studies. In a longitudinal analysis, Herbert and colleagues found that depression was a crucial hurdle for women with fertility issues to seek medical advice^25^. Apart from Herbert and colleagues' study, few longitudinal studies have incorporated the impacts of sociodemographic factors such as income, geographical location, marital status, and lifestyle factors such as tobacco and alcohol intake and Body Mass Index (BMI) on fertility status and mental health.

Therefore, this study aims to fill this knowledge gap by presenting a longitudinal analysis of the association of infertility and mental health in Australian women, taking into account sociodemographic and lifestyle factors. From a broader needs perspective, our study is in alignment with one of the five priority areas (maternal, sexual, and reproductive health) of the recently launched National Women's Health Strategy 2020 to 2030 by the Department of Health, Australia^26^, which highlights the growing importance of this issue and the need for this project.

## Methods

### Data source

This study used data over 18 years from survey 2 (22–27 years of age in 2000) to survey 8 (40–45 years of age in 2018) of the 1973–78 birth cohort of the Australian Longitudinal Study on Women's Health (ALSWH)^27^.

Data collection for the study's survey 1 commenced in 1996 when women were aged 18–23 years old. The participants were randomly selected from the national health insurer's database (Medicare Australia) and were broadly representative of women of a similar age in the Australian population^27^. As data on fertility status was only collected from Survey 2 (1996) onwards, Survey 1 has been excluded from this study (details below). Questionnaires, reports and other research outcomes are available on the ALSWH website (http://www.alswh.org.au), and more details have been published elsewhere^27^. All methods were performed in accordance with the relevant guidelines and regulations.

### Data sampling

**Figure 1:**
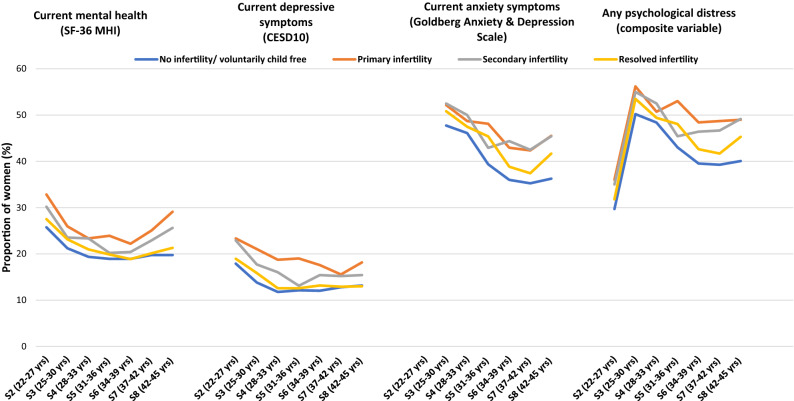
Proportion of 6580 women reporting poor mental health, anxiety, depression and any psychological distress at each survey, according to the fertility status.

The flow chart in Fig. 1 displays the sampling procedure, along with the inclusion and exclusion criteria. We included data from 6582 of the 14,247 women who had completed at least one survey from Survey 2 to Survey 8. We excluded data from women (1) who had not completed Survey 2, which was considered the baseline for this analysis (n = 4559); (2) those with three or more missing surveys (n = 3018); and (3) those with missing information about fertility status on three or more surveys (n = 88) (discussed further below).

#### Defining 'fertility status' using multiple variables approach

We used the following variables related to 'infertility' and 'child status' to determine the women's 'fertility status' overtime:

##### Infertility

From Survey 2 onwards, women were asked if they and their partner (current or previous) ever had problems with infertility, defined as having tried unsuccessfully to get pregnant for 12 months or more. Women selected one of the four response options: never tried to get pregnant; no problem with infertility; yes, but have not sought help/treatment; and yes, and have sought help/treatment.

##### Number and timing of child(ren)

We matched the data from eligible participants with an additional ALSWH data set of "child data" including the number and the presence or absence of children.

##### Fertility status

We used the above variables to determine the women's fertility status overtime in four mutually exclusive categories:

1. *No infertility issues/voluntarily childfree*: no infertility problems reported on any survey and no reported births in the 'child data'/ pregnancies
2. *Primary infertility*: infertility problems reported at one or more surveys and no children at all surveys
3. *Secondary infertility*: infertility problems reported at one or more surveys with child(ren) born before reported infertility
4. *Resolved infertility*: infertility problems at one or more surveys but the child(ren) born after they reported infertility

In further analysis, the category of 'No infertility/ voluntarily child free' was chosen as the reference category.

#### Measurement of mental health

Presence and levels of reported anxiety and depressive symptoms were assessed by the three validated measures described below:

##### Current mental health

The SF-36 Mental Health Index (SF-36 MHI) is a five-item subscale of the SF-36 quality of life measure^28^ which was collected at all survey rounds. The five items were used to generate a score of 0 to 100 with higher scores indicating better mental health^29^. We applied the commonly used cut-point of ≤ 52 to categorise women as reporting psychological distress at each survey^30,31^. Previous research has established this cut point to be conservatively within the bounds of a clinically meaningful indicator of psychological distress^29^.

##### Current depressive symptoms

The 10-item version of the Center for Epidemiological Studies Depression Scale (CESD-10) scores (ranging from 0 to 30) were used to measure current depressive symptoms at each survey^28^. A cut-point of ≥ 10 was indicative of a potential clinical diagnosis of depression^32^.

##### Current anxiety symptoms

The Goldberg Anxiety and Depression Scale (GADanx) anxiety subscale was included in questionnaires from Survey 3 onwards and was used to measure current symptoms indicative of an anxiety disorder. A score greater than five indicates a potential anxiety disorder^33^.

Additionally, we created a composite variable for 'any psychological distress'. Women were identified as having 'any psychological distress' if in any survey, they (1) self-reported anxiety or depression in the last 3 years or (2) their scores on any of the above three validated measures were above or below the respective cut-points described above.

### Explanatory variables

We included sociodemographic, chronic health and behavioural factors in the analysis to check and adjust for potential confounding in the association between infertility and mental health.

These were

1. Demographic factors: the highest level of qualification (no education, school certificate, trade/certificate/diploma and higher education), marital status (partnered, not partnered), area of residence as per the Accessibility/remoteness Index of Australia (ARIA) Plus classification system^34^; paid work status (not in paid work/in paid work); self-reported general health (excellent/good, fair/poor);
2. Chronic health issues including diabetes, heart disease, high blood pressure, asthma, cancer and other major physical illness and smoking status (non-smoker, ex-smoker, current smoker); and
3. Health behavior factors: alcohol consumption (non-drinker, low-risk drinker, high-risk drinker) and BMI (< 18.5, 18.5–24.9, 25–29.9, ≥ 30)

Missing at random data were filled using the 'last observation carried forward (LOCF)' approach to maintain the sample size and reduce the bias caused by the attrition of participants in the study.

### Statistical analysis

We used descriptive statistics to analyse baseline demographics and describe psychological distress. We used longitudinal, repeated measures models utilising the generalised estimating equations (GEE) method in parameter estimation for both univariate and multivariate data modelling to test for the presence of an association between ‘Fertility Status and Psychological distress’ (composite variable). GEE analysis requires some key assumptions to be maintained, such as dependent variable linearly related to the predicters, high number of clusters, and observations independent of each other^35^. ALSWH data fulfilled these assumptions, and, therefore, GEE was considered the analysis of choice. Furthermore, GEE enables us to conduct longitudinal data analysis with dichotomous, categorical, nominal or ordinal variables^35^ and, therefore, was effective for examining the ALSWH data. Each GEE model (described below) was adjusted for time (six survey rounds) and 'fertility status' with 'psychological distress' as the dependent variable. Variables with a *p* value of less than 0.25 in the initial univariate approach were selected and entered into the multivariate models as described below^36^.

- **Model 1:** Adjusted for time and fertility status
- **Model 2:** Adjusted for time and fertility status + demographic factors
- **Model 3:** Adjusted for time and fertility status + demographic factors + common chronic health conditions
- **Model 4:** Fully adjusted for time and fertility status + demographic factors + common chronic health conditions + health behaviour factors.

For health conditions (Model 3 and Model 4), the status of 'No' was the reference category. The exchangeable correlation structure approach was utilised for the multivariate modelling based on a smaller QIC (Quasilikelihood under the Independence model Criterion) statistic. The results were reported using odds ratios and corresponding 95% confidence intervals. SAS 9.4 was used for all analyses.

### Ethics approval

This project was approved by the Human Research Ethics Committees (HREC) of the University of Newcastle and the University of Queensland (The University of Newcastle HREC *EC00144*, ratified by The University of Queensland HREC *EC00456/7*). The ALSWH survey program has ongoing ethical approval from the Human Research Ethics Committees (HRECs) of the Universities of Newcastle and Queensland (approval numbers H-076-0795 and 2004000224, respectively, for the 1973–78 cohort). Informed consent was obtained from all participants. All participants consented to joining the study and were free to withdraw or suspend their participation at any time with no need to provide a reason.

## Results

A total of 6582 women were eligible to be included in the analysis. There was a steady increase in the proportion of women reporting problems with infertility over time. The proportion of women reporting having infertility problems and seeking treatment increased from 1.7% at survey 2 to 19.3% at survey 8, which shows a massive increase in reported infertility (see Table 1).

**Table 1:** Proportion of women' ever’ reporting presence or absence of infertility from Survey 2 to survey 8.

N = 6582					
Surveys	Age (years)	Never tried to get pregnant (%)	No problem with infertility (%)	Yes, had a problem with infertility	
				Not sought help/treatment (%)	Sought help/treatment (%)
Survey 2	22–27	79.4	17.7	1.1	1.7
Survey 3	25–30	65.3	28.5	1.8	4.3
Survey 4	28–33	45.6	43.4	3.3	7.6
Survey 5	31–36	31.0	52.6	3.9	12.3
Survey 6	34–39	22.9	56.6	4.8	15.5
Survey 7	37–41	18.2	59.2	4.8	17.6
Survey 8	40–45	17.0	58.4	5.1	19.3

Table shows the percentages from the original variable from the survey. The question asked was “Have you and your partner (current or previous) ever had problems with fertility—that is, tried unsuccessfully for 12 months or more to get pregnant?”.

Using the definition for ‘fertility status over time’, the four mutually exclusive categories were created (from the original variable). As seen in Table 2, 69.2% of women had no reported infertility problem or were voluntarily childfree. Among the women with some reported infertility problems, the majority (18.11%) had resolved infertility.

**Table 2:** The distribution of mutually exclusive ‘fertility status’ for 6580 women.

Fertility status	N	%
No infertility/voluntarily childfree	4559	69.26
Primary infertility	347	5.27
Secondary infertility	480	7.29
Resolved infertility	1192	18.11

Table 3 compares each fertility status category's demographic, health, and health behaviour factors at baseline survey 2. The majority of the women in all the categories of fertility status had higher education, were not partnered, were working in paid jobs and living in urban areas. Women reported their general health to be excellent or good. Chronic health conditions such as diabetes, heart disease, high blood pressure, asthma and other physical illnesses were reported higher in women with primary infertility as compared to women reporting secondary infertility, resolved infertility or no infertility. Most women were non-smokers, engaged in low-risk alcohol use, and had an acceptable BMI (Table 3).

**Table 3:** Descriptive and baseline profile of 6,582 women, by subgroup according to fertility status using survey 2 as the baseline survey

Variables	Fertility status				Cohort profile
	No infertility/voluntarily child free (%)	Primary infertility (%)	Secondary infertility (%)	Resolved infertility (%)	1973–78 cohort profile (n = 9694)
**Demographic factors**					
**Highest education**					
No education	1.03	0.29	1.67	0.84	1.2
School certificate	28.60	26.51	30.42	27.85	30.7
Trade/Cert/Dip	21.69	23.34	24.17	24.24	23.3
Higher education	45.76	49.97	40.21	43.71	44.8
**Marital status**					
Not partnered	55.06	74.06	49.38	49.24	56.2
Partnered	20.33	16.14	22.08	23.32	43.8
**Area of residence**					
Urban	55.36	60.81	53.96	56.80	66.60
Rural	40.67	34.87	41.25	40.35	31.00
Remote	3.53	3.75	4.38	2.77	2.3
**Paid work**					
Not in paid work	3.49	1.15	2.92	2.01	3.7
In paid work	82.72	89.05	79.58	88.17	96.3
**Health factors**					
**General health**					
Excellent/good	90.50	85.88	85.83	88.93	88.10
Fair/poor	9.32	14.12	13.75	10.99	11.90
**Chronic health issues**					
Diabetes (Y)	0.22	0.58	0.42	0.34	0.30
Heart disease (Y)	0.11	0.29	0.05	0.08	0.10
High BP (Y)	2.52	4.32	3.33	2.77	2.50
Asthma (Y)	10.05	15.85	10.63	10.91	11.10
Cancer (Y)	0.55	2.02	1.88	0.52	0.80
Other major physical illness (Y)	3.18	4.61	4.79	3.52	3.70
**Health behaviour factors**					
**Smoking status**					
Non-smoker	60.67	59.08	54.79	60.82	58.1
Ex-smoker	14.43	10.66	16.46	12.75	14
Current smoker	24.19	28.24	28.13	25.67	27.9
**Alcohol consumption**					
Non-drinker	7.81	6.05	5.21	8.47	8.80
Low-risk drinker	88.35	87.61	88.96	87.84	87.5
High-risk drinker	3.29	6.05	4.38	3.44	3.70
**BMI**					
< 18.5	6.08	5.19	5.00	5.54	7.00
18.5–24.9	60.14	57.35	59.38	61.91	64.00
25–29.9	18.60	17.87	17.29	17.79	19.10
≥ 30	8.91	17.87	10.83	10.15	10.00

Missing values for the demographic, health and health behaviour factors not reported in this table.

Figure 1 presents the proportions of the reported psychological distress variables—SF-36 MHI, CESD10 and Goldberg anxiety and depression scales, along with the psychological distress variable over time. The proportion of women with no infertility reporting poor mental health on the SF-36 MHI decreased and stabilised over time; however, the proportion of women reporting poor mental health with primary infertility gradually increased. Fluctuation in reporting poor mental health was seen among women with secondary and resolved infertility; however, a considerable proportion reported poor mental health by survey 8 (women aged 42–45 years). Considerable fluctuation in the proportion of women reporting current depressive symptoms (CESD10) was seen over time. Compared to Survey 2 and Survey 3, few women reported current anxiety symptoms over time. Nearly 50% of women with primary and secondary infertility met the criteria for any psychological distress using the composite psychological distress variable. Women without infertility issues and resolved infertility also had a high proportion of women (40% and 45% respectively) reporting any psychological distress.

The results of the study showed that the odds of reporting psychological distress significantly increased in all models with time (see Table 4). The effect of fertility status on the psychological distress was significant for women with primary and secondary infertility (*p* < 0.001) and for women with resolved infertility (*p* < 0.001), as compared to women without infertility problems or voluntarily child free. In the fully adjusted GEE model, many covariates made significant independent contributions to psychological distress. For example, women who were partnered, reported having diabetes, high blood pressure, asthma, major physical illness, were currently smoking or were ex-smoker, were high-risk drinkers, and reported either with BMI < 18.5 ≥ 30 had higher odds of reported psychological distress over time (*p* < 0.05), compared to women in reference categories. Women who were in paid work were less likely to report psychological distress over time (*p* < 0.05). Few variables such as area of residence, heart disease, cancer was not statistically significant in the adjusted models (Table 4).

**Table 4:** Association between ‘fertility status’ and ‘any psychological distress’ over time—repeated measures approach using GEE.

	Model 1^1^		Model 2^2^		Model 3^3^		Fully adjusted Model 4^4^	
	OR (95% CI)	*p*	OR (95% CI)	*p*	OR (95% CI)	*p*	OR (95% CI)	*p*
**Survey periods**								
Survey 2 (22–27 years)	1		1		1		1	
Survey 3 (25–30 years)	2.81 (2.64; 2.98)	< 0.0001	2.97 (2.74; 3.22)	< 0.0001	3.20 (2.94; 3.49)	< 0.0001	3.27 (2.99; 3.58)	< 0.0001
Survey 4 (28–33 years)	2.41 (2.26; 2.56)	< 0.0001	2.65 (2.43; 2.90)	< 0.0001	2.90 (2.63; 3.20)	< 0.0001	2.98 (2.70; 3.30)	< 0.0001
Survey 5 (31–36 years)	2.10 (1.98; 2.24)	< 0.0001	2.02 (1.88; 2.18)	< 0.0001	2.21 (2.04; 2.40)	< 0.0001	2.36 (2.17; 2.57)	< 0.0001
Survey 6 (34–39 years)	1.72 (1.61; 1.83)	< 0.0001	1.65 (1.54; 1.78)	< 0.0001	1.74 (1.60; 1.88)	< 0.0001	1.86 (1.71; 2.03)	< 0.0001
Survey 7 (37–42 years)	1.96 (1.84; 2.09)	< 0.0001	1.95 (1.80; 2.10)	< 0.0001	2.01 (1.85; 2.18)	< 0.0001	2.13 (1.95; 2.33)	< 0.0001
Survey 8 (42–45 years)	2.20 (2.06; 2.35)	< 0.0001	2.17 (2.01; 2.34)	< 0.0001	2.22 (2.04; 2.41)	< 0.0001	2.35 (2.14; 2.56)	< 0.0001
**Fertility status**								
No infertility/no child voluntarily	1		1		1		1	
Primary infertility	1.39 (1.19; 1.61)	< 0.0001	1.35 (1.16; 1.59)	0.0002	1.26 (1.08; 1.47)	0.0040	1.24 (1.06; 1.45)	0.0076
Secondary infertility	1.32 (1.16; 1.51)	< 0.0001	1.34 (1.16; 1.54)	< 0.0001	1.28 (1.11; 1.47)	0.0006	1.27 (1.10; 1.46)	0.0011
Resolved infertility	1.13 (1.03; 1.23)	0.0065	1.13 (1.03; 1.24)	0.0074	1.15 (1.05; 1.26)	0.0033	1.15 (1.05; 1.26)	0.0026
**Demographic factors**								
**Marital status**								
Not partnered			1		1		1	
Partnered			1.14 (1.07; 1.22)	< 0.0001	1.11 (1.04; 1.18)	0.002	1.06 (0.99; 1.13)	0.0900
**Area of residence**								
Urban			1		1		1	
Rural			0.98 (0.92; 1.05)	0.6000	0.98 (0.91; 1.04)	0.46	0.95 (0.89; 1.01)	0.12
Remote			0.84 (0.70; 1.02)	0.0800	0.90 (0.73; 1.10)	0.31	0.86 (0.70; 1.07)	0.18
**Paid work**								
Not in paid work			1		1		1	
In paid work			0.85 (0.79; 0.91)	< 0.0001	0.92 (0.85; 0.99)	0.0291	0.93 (0.86; 1.00)	0.0600
Chronic health issues*								
Diabetes (yes)					1.49 (1.12; 1.97)	0.0059	1.43 (1.07; 1.91)	0.0151
Heart disease (yes)					0.85 (0.50; 1.46)	0.56	0.84 (0.50; 1.43)	0.53
High blood pressure (yes)					1.37 (1.19; 1.57)	< 0.0001	1.28 (1.11; 1.48)	0.0006
Asthma (yes)					1.30 (1.17; 1.43)	< 0.0001	1.29 (1.17; 1.43)	< 0.0001
Cancer (yes)					1.11 (0.88; 1.40)	0.39	1.08 (0.86; 1.37)	0.51
Other major physical illness (yes)					1.63 (1.46; 1.82)	< 0.0001	1.62 (1.45; 1.81)	< 0.0001
**Health behaviour factors**								
**Smoking status**								
Non-smoker							1	
Ex-smoker							1.31 (1.21; 1.42)	< 0.0001
Current smoker							1.73 (1.57; 1.90)	< 0.0001
**Alcohol consumption**								
Non-drinker							1	
Low-risk drinker							1.08 (0.97; 1.20)	0.17
High-risk drinker							1.51 (1.27; 1.78)	< 0.0001
**BMI**								
18.5–24.9							1	
< 18.5							1.44 (1.21; 1.72)	< 0.0001
25–29.9							1.01 (0.94; 1.08)	0.82
≥ 30							1.20 (1.10; 1.31)	< 0.0001

^1^Model 1: Adjusted for time and fertility status.

^2^Model 2: Adjusted for time and fertility status + demographic factors.

^3^Model 3: Adjusted for time and fertility status + demographic factors + Common chronic health conditions.

^4^Model 4: Fully adjusted for time and fertility status + demographic factors + Common chronic health conditions + health behaviour factors.

*For health conditions, the status of 'No' is the reference category.

## Discussion

This research project assessed the longitudinal associations between fertility status and psychological distress over time among Australian women. To the best of our knowledge, this study is one of the first in Australia to explore these longitudinal associations over 18 years of data. The findings of the longitudinal analysis show that fertility status is an enduring condition that has a significant association with mental health outcomes among our sample of Australian women of reproductive age followed for 18 years from their 20 s to their early 40 s. The study also indicated that mental health impact remains highly substantial among the women who reported primary or secondary infertility and those who reported ‘resolved infertility’. This finding reveals the long-term impact of fertility issues and problems on women.

These findings are corroborated by previous research, which showed that infertility impacts women’s overall self-esteem, confidence, and performance^37^. A previous longitudinal study by Herbert et al. on Australian women’s health data found that out of 5936 women, 1031 women with infertility reported higher odds of self-reported depression than 4905 women who were not suffering from infertility^25^. Herbert et al. also indicated that women with depression and depressive symptoms were less likely to utilise healthcare to treat infertility, which may not resolve infertility^25^. Similarly, other researchers have demonstrated unresolved infertility's adverse long-term mental health impact through several longitudinal studies in Italy, Canada, Denmark, Australia, and Germany^7,16,38,39^.

The desire to have a child is common for many couples and individuals and is emphasised by continued cultural and societal norms^9,40^. In several pronatalist cultures, childlessness is associated with the stigma of disgrace, shame, and societal shunning, in addition to marital discord^41,42^. In many countries, cultural and societal pressure demands women to have at least one biological child or face discrimination, stigmatisation, and ostracism^42,43^. Infertility/subfertility is also associated with higher intimate partner violence^44^. Couples, therefore, choose to remain silent and avoid the anxiety associated with the stigma of infertility and its treatment^45^. Galhardo et al. recorded higher levels of depression and a sense of shame in couples diagnosed with infertility compared to those with no known diagnosis of infertility and adoption-ready couples with a diagnosis of infertility^46^. The researchers also found that couples with the diagnosis of infertility resorted to negative coping mechanisms (avoidant) in comparison to the rational styles (acceptance) of coping reported by adoption-ready couples^46^.

The impact of infertility on mental health may be due to various intersecting reasons. These include the slow and unpredictable success rates of infertility treatment (Chambers, Sullivan, & Ho, 2006), leading to added stress and anxiety, particularly in socioeconomically disadvantaged groups^47^. In many countries, assisted reproductive technology (ART) is expensive, and ART services are either partially included or not included under the government primary healthcare package, nor is it entirely covered by private health insurance^1^. Therefore, the financial barrier to access ART services adds to the infertility treatment-related stress^1^.

In this study, partnered women reported more psychological distress than non-partnered women. This difference may be linked to the socio-cultural aspects of infertility, where in some cultures, childbearing is considered an essential component of married life and is viewed as a symbol of social status^21,22^. Alternatively, it may be explained by understanding how couples cope with infertility compared to non-partnered individuals. Two longitudinal studies have highlighted that couples with infertility that use active and passive coping strategies (such as avoidance) have higher psychological distress compared to couples engaged in meaning-based coping strategies (problem-focused strategies, motivation, and optimism)^45,48^.

In this study, women in paid employment reported significantly lower psychological distress (Chambers et al., 2013). This finding is not surprising since paid employment gives financial stability and helps couples with higher socioeconomic status have more resources to seek and utilise infertility treatment^47^.

We found that having an underweight BMI or being obese was significantly associated with psychological distress. Scott et al. (2008) analysed 62,277 people in the world mental health surveys and reported that high BMI was modestly associated with mental health disorders in women^49^. Previous studies have highlighted that obesity or being underweight, both extremes can adversely affect fertility^50–52^. Esmaeilzadeh et al. (2013) have also found that women with infertility experience had a 4.8-fold increased risk of obesity compared to women without infertility^53^. Therefore, both high or extremely low BMI should be considered risk factors during mental health and infertility treatment.

Further, the analysis of behavioural health factors such as the prevalence of smoking and alcohol were significantly higher in women reporting psychological distress. Apart from anxiety, there was a significant prevalence of certain chronic illnesses such as diabetes, high blood pressure, asthma, and other major physical illnesses in women reporting psychological distress. Herbert et al. have also reported similar findings (2010). Although our study did not find a significant association between fertility status and psychological distress in women with cancers, previous studies have highlighted that fertility-related psychological distress is prevalent in cancer patients and survivors^54^. In another extensive epidemiological review that analysed 82 articles, Direkvand-M et al. iterated that modifying lifestyle factors such as smoking, alcohol and physical activity, and early diagnosis and management of chronic diseases can significantly help to improve the fertility status in women^55^. Lifestyle risk factors such as smoking, alcohol, unhealthy dietary habits and physical inactivity are responsible for several chronic diseases. The significant findings regarding the lifestyle factors call for a holistic approach during infertility treatment, including awareness and promoting behavioural changes in diet, physical activity, smoking, and alcohol use.

Our study had a few expected limitations. Firstly, the gender differences could not be analysed. Although men with infertility also suffer from poor mental health outcomes^16,56^, nationally representative men-only longitudinal data on infertility is unavailable in Australia. Future researchers would focus on assessing gender differences longitudinally. Secondly, we assumed that data was ‘missing at random’, and these missing values were filled in using the last observation carried forward approach that may have caused analytic bias; however, we tried to minimise this possibility by only imputing missing at random observations and by excluding women with three or more missing surveys. Despite a rigorous participant recruitment strategy, the ALSWH study has lower representation from minority groups, Indigenous population, refugees, and non-English speaking migrants. This low representation from diverse community groups is an issue that needs to be addressed at multiple levels starting from the national policy and is, therefore, beyond the scope of the study. We were also unable to include infertility treatments in our assessments as these are not covered by the Medicare (healthcare access scheme for Australians and some visa holders) and hence not a part of the survey and/or linked administrative data. The other limitation of the study was that we could not include clinical conditions related to infertility, such as, polycystic ovarian syndrome (PCOS) or endometriosis. Apart from affecting fertility, PCOS is significantly associated with severe mental health distress, body dissatisfaction and eating disorders^57,58^. Further studies should be conducted to understand the relationship of clinical conditions that affect fertility status such as PCOS on mental health distress.

However, the study has several conceptual, methodological, and analytical strengths that have helped us understand the importance of using an integrated approach to treat patients with infertility. We conducted an extensive literature review to understand the gaps in the literature and identify the strengths of different longitudinal studies. ALSWH is the largest and longest-running survey and provides an in-depth insight into various aspects of women’s health in Australia. The longitudinal approach gives an opportunity to follow up the trends of fertility status and mental health in women’s health for 18 years. We independently assessed three validated measures of psychological distress and created a composite score to define psychological distress at each survey; defined fertility status in four mutually exclusive categories by using survey and child and birth data to capture all the relevant details. With these rich data over seven surveys and covering almost 18 years, Generalised Estimating Equation was a robust technique to assess the longitudinal associations.

## Conclusion

This study highlighted that infertility is a multidimensional stressor causing anxiety, stress, and depression with long-lasting mental health consequences and makes a strong case for infertility as a strategic public health priority. The study has important implications for implementing the current Australian National women’s health strategy 2020–2030. The implication includes improving the assessment of infertility issues as well as equitable access and affordability of infertility treatment for all groups of population. The assessment of infertility should consist of a comprehensive approach beyond clinically focused management and plans for risky lifestyle behaviours such as smoking and alcohol use. Regular mental health screening should be conducted for all patients, especially in women with primary, secondary, or resolved infertility with easy and accessible access to mental health support to protect women from long-term mental health impacts.

Infertility is rapidly emerging as a significant global health issue. Human overpopulation is causing over consumption of natural resources and causing climate change. The global warming crisis and environmental pollutants are increasing the burden of diseases and a rise in fertility issues. Environmental health affects human health and therefore, we should also draw attention to on the broader issues such as climate change and overpopulation.

## Data Availability

The Australian Government Department of Health owns ALSWH survey data and due to the personal nature of the data collected, release by ALSWH is subject to strict contractual and ethical restrictions. Ethical review of ALSWH is by the Human Research Ethics Committees at The University of Queensland and The University of Newcastle. De-identified data are available to collaborating researchers where a formal request to use the material has been approved by the ALSWH Data Access Committee. The committee is receptive of requests for datasets required to replicate results. Information on applying for ALSWH data is available from https://alswh.org.au/for-data-users/applying-for-data.
